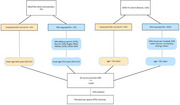# Investigation of enlarged perivascular spaces as a biomarker for head impact exposure across two independent cohorts

**DOI:** 10.1002/alz70856_096740

**Published:** 2025-12-24

**Authors:** Suzie Kamps

**Affiliations:** ^1^ Amsterdam Neuroscience, Neurodegeneration, Amsterdam, Netherlands; Alzheimer Center Amsterdam, Neurology, Vrije Universiteit Amsterdam, Amsterdam UMC location VUmc, Amsterdam, Netherlands

## Abstract

**Background:**

Repetitive Head Impacts (RHI), common in contact sports, are associated with an increased risk of neurodegenerative diseases. Exact mechanisms underlying this relationship are unknown. For Traumatic Brain Injury (TBI), it has been repeatedly suggested that impaired brain clearance could be a potential mechanism for accumulation of tau proteins in the brain. It remains largely unknown whether similar impairments in clearance through the perivascular transport system occur with RHI exposure and if this contributes to the development of dementia symptoms. Impaired perivascular clearance can be indicated by enlargement of perivascular spaces (PVS). We aimed to quantify PVS volumes to investigate whether RHI is associated with enlarged PVS (ePVS). We set out to cross‐validate results by investigating this in two independent cohorts exposed to RHI.

**Method:**

We included from two cohorts, NEwTON (Amsterdam, NL) and SAVE‐HI (Boston, USA). Both cohorts include individuals exposed to RHI due to long term contact sports participation (*N*_Amsterdam_ = 75, 10% female; *N*_Boston_ = 200, 20% female) and age‐and‐sex matched healthy, unexposed controls (*N*_Amsterdam_ = 40, 17.5% female; *N*_Boston_ = 100, 20% female). All participants underwent structural 3D brain MRI, cognitive testing, plasma sampling, and questionnaires for sleep quality. MRI scans are processed through a validated pipeline to calculate PVS volumes in the white matter. We perform analyses of variance (ANOVA) to test differences in PVS volumes between the groups (RHI vs. unexposed). Regression models are employed to measure associations between PVS volumes, neurocognitive functioning, sleep quality and the degree of RHIexposure (in years). As sensitivity analyses, these analyses are repeated after stratifying individuals for amyloid positivity based on plasma *p*‐tau217 concentrations.

**Result:**

Data is expected to be fully processed by April of 2025. We expect individuals exposed to RHI to have larger PVS volumes than unexposed controls. Further, PVS volume is expected to be related to longer RHI exposure and worse outcomes for neurocognitive functioning and sleep quality. Finally, stronger associations are anticipated in individuals that are amyloid positive based on plasma *p*‐tau217.

**Conclusion:**

Identification of easily accessible biomarkers for RHI exposure, such as ePVS, is important to recognize individuals at risk for neurodegeneration following their contact sports participation.